# Distal Pancreatectomy with Celiac Axis Resection: Systematic Review and Meta-Analysis

**DOI:** 10.3390/cancers13081967

**Published:** 2021-04-19

**Authors:** Giuseppe Nigri, Niccolò Petrucciani, Elena Belloni, Alessio Lucarini, Paolo Aurello, Francesco D’Angelo, Salomone di Saverio, Alessandro Fancellu, Giovanni Ramacciato

**Affiliations:** 1 Department of Medical and Surgical Sciences and Translational Medicine, Sapienza University of Rome, Sant’Andrea Hospital, 00189 Rome, Italy; niccolo.petrucciani@uniroma1.it (N.P.); elena.belloni@uniroma1.it (E.B.); alessio.lucarini@uniroma1.it (A.L.); paolo.aurello@uniroma1.it (P.A.); francesco.dangelo@uniroma1.it (F.D.); giovanni.ramacciato@uniroma1.it (G.R.); 2 Department of Surgery, Cambridge University Hospitals NHS Foundation Trust, Cambridge CB2 0QQ, UK; salo75@inwind.it; 3 Department of Medical, Surgical and Experimental Sciences, University of Sassari, 07100 Sassari, Italy; afancel@uniss.it

**Keywords:** pancreatic cancer, borderline resectable, vascular reconstruction

## Abstract

**Simple Summary:**

The literature is conflicting regarding the feasibility and survival outcomes of distal pancreatectomy with celiac axis resection (DP-CAR), although this procedure, in selected cases, represents the only therapeutical option for patients with locally advanced pancreatic cancer. The available studies often include small surgical populations, and there are important variations in the inclusion criteria and pre- and post-operative treatment. The purpose of this study was to provide an overview of the literature of the last 15 years, to evaluate the efficacy and the clinical safety of this procedure. This could help physicians in the choice of a multidisciplinary targeted therapeutical plan for patients. The combination of neoadjuvant chemo/radiochemotherapy and demolitive surgeries such as DP-CAR could have a role in changing the survival outcomes of patients with locally advanced pancreatic adenocarcinoma.

**Abstract:**

Background: Major vascular invasion represents one of the most frequent reasons to consider pancreatic adenocarcinomas unresectable, although in the last decades, demolitive surgeries such as distal pancreatectomy with celiac axis resection (DP-CAR) have become a therapeutical option. Methods: A meta-analysis of studies comparing DP-CAR and standard DP in patients with pancreatic adenocarcinoma was conducted. Moreover, a systematic review of studies analyzing oncological, postoperative and survival outcomes of DP-CAR was conducted. Results: Twenty-four articles were selected for the systematic review, whereas eleven were selected for the meta-analysis, for a total of 1077 patients. Survival outcomes between the two groups were similar in terms of 1 year overall survival (OS) (odds ratio (OR) 0.67, 95% confidence interval (CI) 0.34 to 1.31, *p* = 0.24). Patients who received DP-CAR were more likely to have T4 tumors (OR 28.45, 95% CI 10.46 to 77.37, *p* < 0.00001) and positive margins (R+) (OR 2.28, 95% CI 1.24 to 4.17, *p* = 0.008). Overall complications (OR, 1.72, 95% CI, 1.15 to 2.58, *p* = 0.008) were more frequent in the DP-CAR group, whereas rates of pancreatic fistula (OR 1.16, 95% CI 0.81 to 1.65, *p* = 0.41) were similar. Conclusions: DP-CAR was not associated with higher mortality compared to standard DP; however, overall morbidity was higher. Celiac axis involvement should no longer be considered a strict contraindication to surgery in patients with locally advanced pancreatic adenocarcinoma. Considering the different baseline tumor characteristics, DP-CAR may need to be compared with palliative therapies instead of standard DP.

## 1. Introduction

Pancreatic cancer is the fourth most common malignancy in the United States, with a 5 year survival rate ranging around 6% [1]. More than 75% of pancreatic adenocarcinomas located at the body/tail of the pancreas are unresectable at the time of diagnosis. The most frequent reasons are major vascular invasion and liver and peritoneal metastasis [2,3,4]. Pancreatic cancer surgery has had an important development in the last decades. An increase in surgical safety has been documented, and a paradigm shift has occurred when it comes to vascular resection. Venous resection is no longer considered a contraindication to surgery and it is routinely performed by trained pancreatic surgeons [5,6,7,8,9,10]. Arterial resections still represent an important practical limit and are associated with high morbidity and mortality, as reported by a systematic review and meta-analysis by Mollberg et al. in 2011 [11]. Clear indications are given regarding the management of pancreatic cancer, stratifying different categories of surgical resectability as “resectable”, “borderline resectable” and “unresectable” [5,12,13]. When it comes to pancreatic body/tail cancers, distal pancreatectomy with splenectomy (DP-S) is the standard of care, but the diagnosis occurs frequently at an advanced stage, where a resection strategy is not indicated due to celiac axis (CA) or common hepatic artery (CHA) involvement [14]. Distal pancreatectomy with en-bloc celiac axis resection (DP-CAR) [15] may provide an alternative to chemo- and/or radiotherapy strategies. However, few studies regarding DP-CAR are present in the literature and different data from different studies are inconsistent and/or controversial [16,17,18,19,20,21,22,23]. Questions are still open as to whether DP-CAR compared to DP or palliative treatment is safe, effective and can guarantee survival benefits or improvement in quality of life (QoL).

The first evidence in the literature for the CA resection without reconstruction was reported for a case of gastric cancer requiring total gastrectomy by Appleby in 1953 [24]. Nowadays, arterial resection of the CA has been translated to DP, in a procedure named DP-CAR [25,26]. This technique has been debated over the years due to its technical difficulty and its morbidity and mortality. Theoretically, it is a valid technique in terms of vascular supply, with the superior mesenteric artery, pancreatoduodenal arcades and gastroduodenal artery being able to provide enough vascular inflow for the hepatobiliary system and the stomach [27]. Practically, ischemic complications continue to be a major concern for this technique [28]. The vascular supply is not the only concern with DP-CAR—the dissection of the neural plexus around the CHA and/or CA may lead to severe diarrhea and malnutrition [28], which some authors have reported controlling with medication [29].

DP-CAR is currently reserved to limited and selected cases in high-volume centers and it is not proposed as a routine treatment option for patients with locally advanced pancreatic cancer. The largest series ever published to our knowledge included 191 patients, covering 16 years and twenty-three institutions from 14 countries [30]. Many other studies—mostly case-series—are documented but comparable data cannot be retrieved. Therefore, the best way to gather different data is through a meta-analysis. Our aim is to determine whether DP-CAR is a safe and feasible procedure.

## 2. Materials and Methods

### 2.1. Methods

The literature search and data analysis were conducted following the recommendations from the Preferred Reporting Items for Systematic Reviews and MetaAnalyses (PRISMA) statement [31].

### 2.2. Literature Search

A systematic literature search was conducted in November 2020 using PubMed, Web of Sciences (WOS), and Scopus databases to identify studies published after 2005, reporting surgical outcomes of distal pancreatectomy with celiac axis resection (DP-CAR) performed for pancreatic cancer. The following keywords were used and combined for the search: arterial resection, superior mesenteric artery, pancreatic carcinoma, celiac artery, celiac axis resection, distal pancreatectomy, modified Appleby, DP-CAR, pancreatic carcinoma, pancreatectomy. References of relevant articles were reviewed. Only articles in English were considered (Table S1). Articles were examined and the following data were extracted: first author, year of publication, study design, number of subjects, patient characteristics, intraoperative outcomes, postoperative outcomes, and survival outcomes.

### 2.3. Data Extraction

The following data were collected, when available, in all studies:

- Patients characteristics: age, gender, neoadjuvant chemotherapy, number of patients undergoing preoperative biliary drainage for obstructive jaundice.
- Tumor characteristics: stage according to AJCC, T stage according to the TNM, tumor size, grade of differentiation (well, moderate, and poor), perineural invasion and nodal status.
- Operation-related outcomes: operative time, blood loss, rates of transfusion, status of resection margins (positive versus negative), postoperative morbidity
- Duration of postoperative hospital stay.
- 30 day mortality, overall and specific postoperative morbidity: postoperative complications >3, defined also according to the Clavien–Dindo classification [32], post-operative pancreatic fistula (POPF) [33], bile leak, delayed gastric emptying (DGE) [34], hemorrhage, rates of blood transfusions, rates of reoperations, number of patients receiving adjuvant chemotherapy.
- Survival outcomes including 1, 3, and 5 year overall survival (OS) and disease-free survival.

### 2.4. Inclusion Criteria

All studies with original data regarding surgical and survival outcomes of DP-CAR in patients with pancreatic carcinoma were included in our analysis. Case-control studies with a control group consisting of patients undergoing standard DP were included in the meta-analysis, while studies without a control group were evaluated for the systematic review. When a patient cohort was reported more than once by the same institution or author, the most recent article was included. The quality of the included studies was assessed by two authors independently (E.B. and A.L.), using the Newcastle Ottawa Scale [35].

Studies with less than five patients were excluded, as well as reviews with no original data, case reports, comments, and letters. Studies that included other types of arterial resection (superior mesenteric artery) or celiac axis resection performed during other surgeries (such as pancreaticoduodenectomy or total pancreatectomy) were excluded when it was impossible to extrapolate data related to patients receiving DP-CAR only. Studies with different outcomes were also excluded. The selection process is summarized in the PRISMA flow chart. 

### 2.5. Outcomes of Interest

All the studies were abstracted for the following relevant data: patient baseline characteristics (age, gender, BMI, type of procedure, intra-operative data (blood loss, operative time) and postoperative outcomes (rate and type of complications, postoperative pancreatic fistula (POPF) [33], mortality, length of stay). The main outcome was the rate of postoperative mortality and morbidity. Secondary outcomes were: postoperative complications >3, defined also according to the Clavien–Dindo classification [32]; rate of pancreatic fistula and delayed gastric emptying (DGE) [34] rate of reoperations; operative time; blood loss; length of hospital stay; rate of positive resection margin and rate of nodal metastases.

### 2.6. Statistical Analysis

Statistical analysis was carried out following the PRISMA statement principles [31]. RevMan software version 5.3 (The Cochrane Collaboration, Software Update, Oxford, UK) was used to perform the meta-analysis. Variables were pooled only if evaluated by three or more studies. For dichotomous variables, odds ratios (ORs) were used as summary measures of efficacy, corresponding to the odds of an event occurring in the arterial resection group compared to the standard DP mini-invasive group. For continuous variables, only data reported as mean ± standard deviation were included. An odds ratio greater than 1 indicated that the probability of an outcome was more likely to occur in the DP-CAR group and was considered statistically significant when *p* < 0.05 and when the 95% confidence interval (CI) did not include the value 1. The Mantel–Haenszel method was used to combine the ORs for outcomes of interest. A random effects model, which is more robust in terms of anticipated heterogeneity, was used. The random effect-weighted mean difference (MD) between groups was used as the summary statistic for continuous variables; 95% confidence intervals were reported. Statistical heterogeneity was evaluated using the I^2^ statistic. I^2^ values of 0% to 25%, 26% to 50%, and >51% were considered to be indicative of homogeneity, moderate heterogeneity and high heterogeneity, respectively. All statistical data were considered significant if *p* < 0.05.

## 3. Results

### 3.1. Included Studies

**Figure 1:**
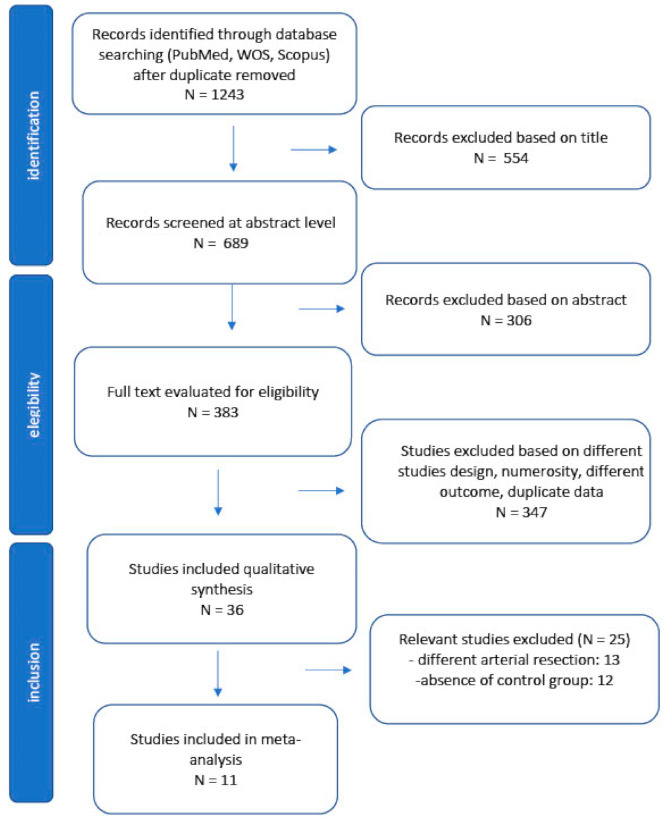
PRISMA flow diagram.

The PRISMA flow diagram is shown in Figure 1. Eleven studies [22,36,37,38,39,40,41,42,43,44,45] comparing DP-CAR and standard DP, published between January 2005 and December 2020, were included in the meta-analysis. On data extraction, there was 100% agreement between the two reviewers. Five studies were conducted in Japan, two in the USA, one study was conducted in China, one in Denmark, one in Germany and one in South Korea. The reports were retrospective studies of comparable patients. No randomized trials were identified. A total of 1077 patients were included, of whom 221 (20%) underwent DP-CAR and 865 (80%) underwent standard DP. The characteristics of the studies are summarized in Table 1. The quality of the studies was assessed using the Newcastle–Ottawa Scale [35] for cohort studies. Of the 13 studies, eight were assessed as high quality (scores between six and seven), and three had a score of five points (Table S2).

### 3.2. Systematic Review

- Patient and Tumor Characteristics

Twenty-four [16,21,22,23,36,37,38,39,40,41,42,43,44,45,46,47,48,49,50,51,52,53,54,55,56] articles were selected for the systematic review for a total of 591 patients (327 male) undergoing DP-CAR. Note that 2.37% (14) were T1, 3.72% (22) T2, 28.1% (166) were T3 and 17.77% (105) T4. Tumor differentiation was as follows: 9.98% (59) G1, 38.4% (207) G2, 14.9% (88) G3. Perineural invasion was registered in 29.3% (173) patients and lymph node positivity was observed in 241 patients (40.8%). 89 (15.0%) patients underwent preoperative hepatic artery embolization.

- Operative Outcomes

The mean operative time varied from 200 to 494 min. In 113 (19.1%) patients, a synchronous venous resection was performed, and direct arterial reconstruction was performed in 32 (5.41%) patients. Positive margins (R+ resections) were observed in 124 patients (20.1%).

- Postoperative Outcomes

Thirty-day mortality was 1.69% (10 patients). Overall morbidity was 39% and 177 (29.9%) patients registered a grade 3 or higher complication. DGE was observed in 37 patients (6.26%), whereas pancreatic fistulae occurred in 199 cases (33.7%). Ischemic gastropathy was observed in 38 patients (6.4%), and two (0.51%) patients experienced kidney failure. Forty-one (6.9%) had postoperative diarrhea, five (0.84%) had postoperative hemorrhage. An increase in postoperative liver transaminase was reported in 12 studies [16,25,28,36,38,40,43,47,50,52,54,55,56]. Hospitalization was very variable, ranging from 7 to 38 days. The rate of reoperation was 3% (18 cases).

Thirteen studies [16,21,28,36,37,38,40,42,46,47,52,54,55], for a total of 441 patients, reported data about neoadjuvant therapy, which was administrated to 248 patients (42% of the total). A total of 358 patients (74.0%) received chemotherapy or chemoradiation adjuvant treatments (data reported in eleven studies [16,21,28,38,41,42,43,44,45,46,54], for a total of 484 patients).

Across all studies, it was reported that the median survival time ranged from 9.7 to 30.9 months, whereas disease-free survival was reported in nine studies [21,28,41,42,45,47,51,54,55] and resulted in a range from 5.2 to 19.4 months. One-year, three-year, and five-year overall survival (OS) were, respectively, 37%, 7.6% and 4.9%.

### 3.3. Meta-Analysis

- Patient and Tumor Characteristics

Patients in the two groups (DP-CAR and DP) were similar with respect to age (mean difference (MD) 1.93, 95% CI −0.12 to 3.97, *p* = 0.07), rates of male gender (OR 0.97, 95% CI 0.71 to 1.33, *p* = 0.85) and use of neoadjuvant therapy (OR 3.19, 95% CI 0.98 to 10.35, *p* = 0.05). 

The distribution of T1 category did not differ (T1: OR 0.68, 95% CI 0.33 to 1.40, *p* = 0.29), whereas T2 and T3 tumors were more represented in the DP group (T2: OR 0.38, 95% CI 0.18 to 0.83, *p* = 0.01; T3: OR 0.45, 95% CI 0.27 to 0.76, *p* = 0.003) and T4 tumors were more represented in the DP-CAR group (OR 28.45, 95% CI 10.46 to 77.37, *p* < 0.00001). Groups were similar with respect to tumor differentiation (well differentiated: OR 0.70, 95% CI 0.18 to 2.75, *p* = 0.61; moderately differentiated: OR 1.06, 95% CI 0.56 to 2.00, *p* = 0.87; poorly differentiated: OR 1.61, 95% CI 0.57 to 4.51, *p* = 0.37). Perineural invasion (PNI) was significantly higher in the tumors of the DP-CAR group (OR 2.13, 95% CI 1.18 to 3.85, *p* = 0.01). Lymph node positivity was more frequently observed in the DP-CAR group (OR 1.56, 95% CI 1.06 to 2.31, *p* = 0.02) (Table 2).

- Operative Outcomes

**Figure 2:**
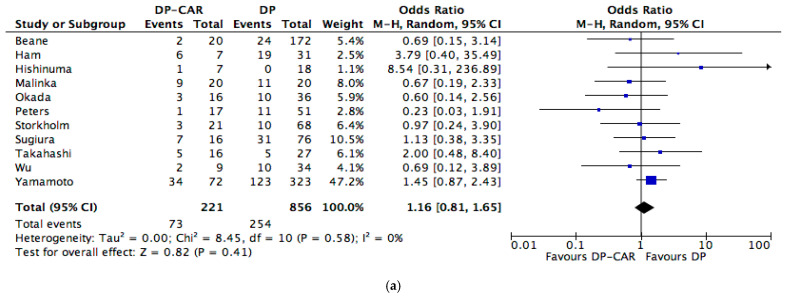

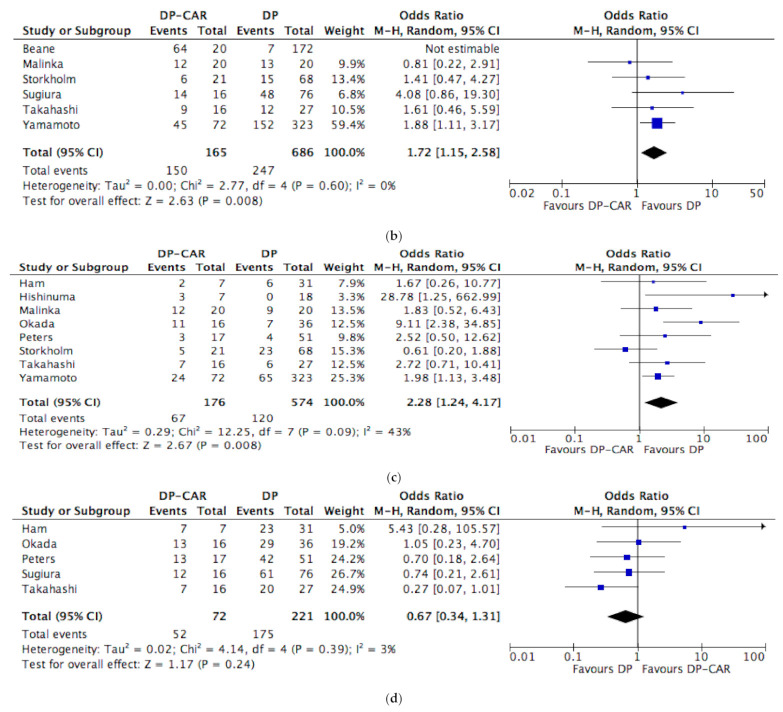
Meta-analysis of post-operative outcome; (**a**) post-operative pancreatic fistula; (**b**) post-operative morbidity; (**c**) positive margins; (**d**) 1-year overall survival.

The rate of synchronous venous resection was higher in the DP-CAR group (OR 7.78, 95% CI 1.70 to 35.61, *p* = 0.008). Blood loss was statistically higher in the DP-CAR group (MD 66.75, 95% CI 26.83 to 106.67, *p* = 0.001) and operative time was longer in the DP-CAR group (MD 78.79, 95% CI 36.73 to 120.85, *p* = 0.002). Rates of positive margins (R+ resections) (OR 2.28, 95% CI 1.24 to 4.17, *p* = 0.008) were higher in the DP-CAR group (Figure 2c).

- Postoperative Outcomes

Rates of postoperative mortality were similar in the two groups (OR 2.55, 95% CI 0.65 to 10.08, *p* = 0.18), whereas overall complications (OR 1.72, 95% CI 1.15 to 2.58, *p* = 0.008) and complications graded 3 or more (OR 1.80, 95% CI 1.22 to 2.65, *p* = 0.003) were more frequent in the DP-CAR group (Figure 2b). Rates of pancreatic fistula (OR 1.16, 95% CI 0.81 to 1.65, *p* = 0.41) (Figure 2a), delayed gastric emptying (OR 1.59, 95% CI 0.64 to 3.99, *p* = 0.32), postoperative bleeding (OR 0.85, 95% CI 0.28 to 2.62, *p* = 0.78) and reoperations (OR 2.54, 95% CI 0.37 to 17.41, *p* = 0.34) were similar.

Hospital stay did not significantly differ (MD −3.08, 95% CI −13.00 to 6.838, *p* = 0.54). 

Data on adjuvant treatments were available in five studies [36,37,38,40,42]. Among 691 patients for whom this aspect was reported, 514 received adjuvant chemotherapy or chemoradiation: 19.8% (102 patients out of 139) and 80.2% (412 patients out of 552) in the DP-CAR and DP group, respectively. 

Survival outcomes were similar in terms of 1 year OS (OR 0.67, 95% CI 0.34 to 1.31, *p* = 0.24), whereas long-term results were not suitable for meta-analysis (Figure 2d).

## 4. Discussion

In patients with body/tail pancreatic adenocarcinoma, DP-CAR has been proposed, but its technical difficulty, its morbidity and mortality have been questioned over the years. The most dreaded complications following this surgical technique are ischemic complications. Hepatic ischemia can occur when the proper hepatic artery (PHA) does not receive enough flow after ligation of the common hepatic artery (CHA). Anatomical variations of the hepatic artery should be taken into account. Gastric ischemia may occur due to poor collateral flow after ligation of the left gastric artery and the left gastroepiploic artery (LGEA) [27]. Makari et al. reported an incidence of ischemic complications in almost 90% of patients who underwent DP-CAR [57]. Hirano et al., in order to reduce the ischemic complications, performed preoperative embolization of the CHA to stimulate the collateralization to the hepatobiliary system (via the pancreaticoduodenal arcade) and the stomach (via the right gastric and gastroepiploic arteries) [28].

However, the available literature is still not clear whether DP-CAR is a safe and effective procedure. The results of our meta-analysis demonstrate why DP-CAR is still considered a technically demanding procedure.

Major blood loss and a prolonged operative time, as expected, were described in the DP-CAR group. This is not surprising considering the surgical procedure itself, which requires a vascular resection and—as described by some authors—a vascular reconstruction [27,58]. In addition, a higher rate of positive margins (R+) was documented by our analysis, which is also not surprising considering the tumor characteristics in the DP-CAR group, where a higher rate of pT4, N+, PNI+ and poorly differentiated tumors were observed, implying a more advanced stage in this group. These results, though, must not be seen as a disadvantage of DP-CAR in favor of standard DP. As stated before, the celiac axis resection is an extension to the resectability criteria of body/tail pancreatic adenocarcinomas, and these results should be seen from this perspective.

Postoperative mortality was slightly higher, although not statistically significant, in the DP-CAR group. Considering what was stated before regarding the complexity of DP-CAR, it is not surprising to find a higher rate of overall complications in this group, which grows even more when it comes to complications graded 3 according to the Clavien–Dindo classification system for postoperative complications [32]. In order to reduce the morbidity and mortality related to ischemic problems following a major vascular resection, an embolization of the CHA has been proposed, as mentioned above. This practice has not been widely reported in our data collection, with a total of 89 patients (15%) who underwent this procedure [16,38,40,41,52,55]. The rates of pancreatic fistula, which remains one of the most dreaded complications of pancreatic surgery [59], were slightly higher in the DP-CAR group. This differs from pancreatic head surgery, in which a vascular involvement is associated, due to fibrosis, with a reduced risk of pancreatic fistula [60].

Nowadays, neoadjuvant chemo/radiochemotherapy is becoming—or already is—a gold standard in almost every gastrointestinal cancer such as cancers of the stomach [61,62] and rectum [63,64], and the most recent guidelines also discuss this approach also when it comes to pancreatic adenocarcinomas of the pancreatic head, body or tail [5,6]. When we searched the literature for evidence, when it comes to DP versus DP-CAR, only few studies reported the use of neoadjuvant chemo/chemoradiotherapy. In our meta-analysis, only five out of 11 studies [36,37,38,40,42] reported the use of a neoadjuvant protocol with a non-significant (but on the verge of a *p* = 0.05) difference in favor of the standard DP. Notably, four out of these five studies were published after 2016 (plus one in 2011 [40]). In our systematic review, we instead found eight studies (plus five from the meta-analysis group) reporting data on neoadjuvant protocols [16,21,28,46,47,52,54,55]. Even in this case, these data were reported in newer studies. This probably represents a paradigm shift in the use of neoadjuvant protocols for pancreatic cancer, which has gained significance over recent years, especially for pancreatic head adenocarcinomas infiltrating the portal/superior mesenteric vein requiring duodeno–cefalo pancreatectomy with vascular resection [65,66]. The possibility of achieving an R0 resection has been associated with a better outcome—Ferrone et al. reported in 2015 a 92% R0 resection rate when a neoadjuvant treatment with FOLFIRINOX was administered to patients with locally advanced pancreatic ductal adenocarcinoma [67], and similar data were reported by Yoshya et al. in 2020 [68]. Moreover, the response to neoadjuvant protocols may help identify patients with more aggressive cancers and worse outcomes. The heterogeneity of the available studies on neoadjuvant chemotherapy in borderline-resectable pancreatic adenocarcinoma makes it difficult to draw firm conclusions on its benefit in regard to short- and long-term outcomes, but the pathway seems to tend towards better outcomes when preoperative treatment has been administered [5,69,70]. Considering the limited evidence for this kind of tumor, both ESMO and NCCN guidelines suggest inserting these patients into clinical trials through high-volume centers for pancreatic oncology and surgery [5,6].

Accurate preoperative staging needs to be addressed, with an expert radiologist looking for specific criteria defining the resectability of the neoplasm, as described in the NCCN guidelines [6]. The decision whether to resect upfront or to use a neoadjuvant protocol needs to be discussed in a multi-disciplinary team meeting, which have been shown to improve the management and the outcomes of neoplastic patients [71,72].

Survival data, as mentioned before, were not suitable for our meta-analysis due to the heterogeneity of the reported data across the different studies. Only 1 year follow up was included in our analysis, and similar results between the procedures were obtained.

Longer survival data may result in favor of DP, but as mentioned, DP-CAR is performed for advanced cancer and further results need to be interpreted with this consideration. It is probable that, depending on overall survival (OS) at longer follow-up, DP-CAR patients should not be confronted with standard DP, but with palliative treatment including chemo/radio-therapy or surgical palliation (as gastro-entero anastomosis) and their impact on quality of life.

The limitation of our study is mainly related to the absence of randomized designed studies included in the meta-analysis. Moreover, differences in outcomes might have resulted from variance in inclusion criteria and operative techniques among studies. Between the eleven studies selected, seven had less than 20 patients in the DP-CAR group and only one study included more than 50 arterial resections. Furthermore, only 32 patients had an arterial resection with arterial reconstruction. We compared the overall survival at 1 year follow-up, even though we are aware that it represents a short follow-up period and the two groups differ concerning tumor characteristics; therefore, the 1 year OS results have limited value.

As for gastric cancer, eastern versus western countries’ differences in population, volume and oncological and surgical approaches need to be addressed. For DP-CAR, given the complexity of the surgical procedure, it has been previously demonstrated that survival outcomes are strongly dependent on surgical volume [30]. In particular, different surgical vascular reconstructions were performed (direct anastomosis, venorrhaphy/patch and interposition graft), and to date there is no clear consensus on the ideal method of vascular reconstruction; these differences might have affected some of the results. Despite these limitations, the present work represents a large comparative analysis of surgical and short-term oncological outcomes in patients undergoing major arterial resection for pancreatic adenocarcinoma of the body/tail of the pancreas.

## 5. Conclusions

In this meta-analysis, all relevant studies published within the last 15 years and providing comparative results on patients undergoing DP-CAR were included. Patients undergoing arterial resection experienced a higher rate of morbidity, operating time and blood loss, compared to the control groups. There were no differences in terms of POPF, reoperation rate, and DGE rate. However, celiac axis resections were not associated with significantly higher mortality compared to standard distal pancreatectomy.

Celiac axis involvement should no longer be considered a strict contraindication to surgery in patients with locally advanced pancreatic adenocarcinoma. What needs to be stated is the absolute importance of a careful selection of patients undergoing this type of surgery, the importance of a multidisciplinary approach and the importance of a highly skilled pancreatic surgery team. Patients need to be aware of the high risk of morbidity that a DP-CAR may lead to. Considering different baseline tumor characteristics, DP-CAR may need to be compared with palliative therapies instead of standard DP.

## Figures and Tables

**Table 1:** Studies selected for the meta-analysis, characteristics.

Author	Year	Country	Time Frame	DP-CAR	DP	Age (DP-CAR/DP)	Gender (M/F)	AJCC T (DP-CAR/DP)	Neoadjuvant Therapy (DP-CAR/DP)	Survival Outcome/Months (DP-CAR/DP)
***Beane***	2015	USA	2011–2012	20	172	64/66	6/14	NR	NR	NR
***Ham***	2015	Korea	2000–2014	7	31	58/67.5	3/4	T1: 0/1 T2: 0/3 T3: 4/25 T 4: 4/2	NR	15/25
***Hishinuma***	2007	Japan	1987–2003	7	18	63.8/NR	4/3	T1: 0/NR T2: 0/NR T3: 6/NR T4: 1/NR	NR	19/25
***Malinka***	2020	Germany	2005–2018	20	20	60.75/65.05	11/9	T1/1/3 T2:3/2 T3: 12/15 T4: 2/0	5/4	NR
***Okada***	2012	Japan	2005–2010	16	36	63/68	11/7	NR	NR	25/25
***Peters***	2016	USA	2004–2016	17	51	64.5/67	9/8	T1: 6/13 T2: 3/25 T3: 1/11 T4: 7/1	15/45	20/19
***Storkholm***	2020	Denmark	2013–2017	21	68	62.3/58.3	13/8	T1: 0/5 T2: 3/17 T3: 6/46 T4: 2/0	3/0	24/23.5
***Sugiura***	2017	Japan	2002–2014	16	76	70/71	10/6	NR	NR	17.5/43.1
***Takahashi***	2011	Japan	1993–2010	16	27	65/70	8/16	T1: 0/5 T2: 0/3 T3: 8/19 T4: 8/0	0/0	9.7/30.9
***Wu***	2010	China	2003–2008	9	34	55.6/62.1	4/5	NR	NR	14/15
***Yamamoto***	2017	Japan	2001–2012	72	323	64/66	40/32	T1: 3/27 T2: 0/28 T3: 57/267 T4:12/1	40/56	17.5/28.6

P-CAR: distal pancreatectomy with celiac axis resection; DP: distal pancreatectomy; M: male; F: female; AJCC: American Joint Committee on Cancer; NR: not reported.

**Table 2:** Studies selected for the meta-analysis, surgical outcomes.

Author	Duration of Surgery—Minutes (DP-CAR/DP)	Blood Loss—mL (DP-CAR/DP)	CA 19.9 (DP-CAR/DP)	N+ (DP-CAR/DP)	R+ (DP-CAR/DP)	Perineural Invasion (DP-CAR/DP)	Neoadjuvant Therapy (DP-CAR/DP)
***Beane***	276/207	NR	NR	NR	NR	NR	NR
***Ham***	354	727/300	281.9 (9.0–2961)/86.7 (2.8–2240.4)	4/10	2/6	7/14	NR
***Hishinuma***	NR	NR	NR	4/NR	3/0	NR	NR
***Malinka***	NR	NR	NR	11/10	12/9	8/5	5/4
***Okada***	298/203	1165/700	NR	NR	11/7	NR	NR
***Peters***	400/309	900/525	30.2 (18.4–45.7)/29.1 (23.4–78.0)	7/20	3/4	NR	15/45
***Storkholm***	245.7/144.7	619/562	NR	16/49	5/23	NR	3/0
***Sugiura***	338/263	902/460	279 (1–1739)/230 (2–2857)	NR	NR	NR	NR
***Takahashi***	237/203	702/634	NR	9/11	7/6	NR	0/0
***Wu***	323/225	889/706	NR	NR	NR	NR	NR
***Yamamoto***	384/265	1033/553	108.8 (1.2–37,370)/100.5 (0–36,399)	50/184	24/65	41/131	40/56

N+: lymph node positivity; R+: positive margins; DP-CAR: distal pancreatectomy with celiac axis resection; DP: distal pancreatectomy.

## Data Availability

Data available on request due to restrictions eg privacy or ethical The data presented in this study are available on request from the corresponding author. The data are not publicly available due to confidentiality.

## Supplementary Materials

The following are available online at https://www.mdpi.com/article/10.3390/cancers13081967/s1, Table S1: Studies selected for the meta-analysis, characteristics, Table S2: Studies selected for the meta-analysis, surgical outcomes.

## References

[1] Jemal A., Siegel R., Xu J., Ward E. (2010). Cancer Statistics, 2010. CA A Cancer J. Clin..

[2] Sohn T.A., Yeo C.J., Cameron J.L., Koniaris L., Kaushal S., Abrams R.A., Sauter P.K., Coleman J., Hruban R.H., Lillemoe K.D. (2000). Resected adenocarcinoma of the pancreas?616 patients: Results, outcomes, and prognostic indicators. J. Gastrointest. Surg..

[3] Fabre J.M., Houry S., Manderscheid J.C., Huguier M., Baumel H. (1996). Surgery for left-sided pancreatic cancer. Br. J. Surg..

[4] Lillemoe K.D., Kaushal S., Cameron J.L., Sohn T.A., Pitt H.A., Yeo C.J. (1999). Distal Pancreatectomy: Indications and Outcomes in 235 Patients. Ann. Surg..

[5] Ducreux M., Cuhna A.S., Caramella C., Hollebecque A., Burtin P., Goéré D., Seufferlein T., Haustermans K., Van Laethem J.L., Conroy T. (2015). Cancer of the pancreas: ESMO Clinical Practice Guidelines for diagnosis, treatment and follow-up. Ann. Oncol..

[6] Tempero M.A., Malafa M.P., Al-Hawary M., Asbun H., Bain A., Behrman S.W., Benson A.B., Binder E., Cardin D.B., Cha C. (2017). Pancreatic Adenocarcinoma, Version 2.2017, NCCN Clinical Practice Guidelines in Oncology. J. Natl. Compr. Cancer Netw..

[7] Fancellu A., Petrucciani N., Porcu A., Deiana G., Sanna V., Ninniri C., Perra T., Celoria V., Nigri G. (2020). The Impact on Survival and Morbidity of Portal-Mesenteric Resection During Pancreaticoduodenectomy for Pancreatic Head Adenocarcinoma: A Systematic Review and Meta-Analysis of Comparative Studies. Cancers.

[8] Nigri G., Petrucciani N., Pinna A.D., Ravaioli M., Jovine E., Minni F., Grazi G.L., Chirletti P., Balzano G., Ferla F. (2018). Evolution of pancreatectomy with en bloc venous resection for pancreatic cancer in Italy. Retrospective cohort study on 425 cases in 10 pancreatic referral units. Int. J. Surg..

[9] Ramacciato G., Nigri G., Petrucciani N., Pinna A.D., Ravaioli M., Jovine E., Minni F., Grazi G.L., Chirletti P., Tisone G. (2016). Pancreatectomy with Mesenteric and Portal Vein Resection for Borderline Resectable Pancreatic Cancer: Multicenter Study of 406 Patients. Ann. Surg. Oncol..

[10] Ramacciato G., Nigri G., Petrucciani N., Pinna A.D., Ravaioli M., Jovine E., Minni F., Grazi G.L., Chirletti P., Tisone G. (2017). Prognostic role of nodal ratio, LODDS, pN in patients with pancreatic cancer with venous involvement. BMC Surg..

[11] Mollberg N., Rahbari N.N., Koch M., Hartwig W., Hoeger Y., Büchler M.W., Weitz J. (2011). Arterial Resection During Pancreatectomy for Pancreatic Cancer. Ann. Surg..

[12] Tempero M.A. (2019). NCCN Guidelines Updates: Pancreatic Cancer. J. Natl. Compr. Cancer Netw..

[13] Soloff E.V., Zaheer A., Meier J., Zins M., Tamm E.P. (2017). Staging of pancreatic cancer: Resectable, borderline resectable, and unresectable disease. Abdom. Radiol..

[14] Buchs N.C., Chilcott M., Poletti P.-A., Buhler L.H., Morel P. (2010). Vascular invasion in pancreatic cancer: Imaging modalities, preoperative diagnosis and surgical management. World J. Gastroenterol..

[15] Klompmaker S., Boggi U., Hackert T., Salvia R., Weiss M., Yamaue H., Zeh H.J., Besselink M.G. (2018). Distal Pancreatectomy with Celiac Axis Resection (DP-CAR) for Pancreatic Cancer. How I do It. J. Gastrointest. Surg..

[16] Yoshitomi H., Sakai N., Kagawa S., Takano S., Ueda A., Kato A., Furukawa K., Takayashiki T., Kuboki S., Miyzaki M. (2019). Feasibility and safety of distal pancreatectomy with en bloc celiac axis resection (DP-CAR) combined with neoadjuvant therapy for borderline resectable and unresectable pancreatic body/tail cancer. Langenbeck’s Arch. Surg..

[17] Gong H., Ma R., Gong J., Cai C., Song Z., Xu B. (2016). Distal pancreatectomy with en bloc celiac axis resection for locally advanced pancreatic cancer: A systematic review and meta-analysis. Medicine.

[18] Yamaguchi K., Nakano K., Kobayashi K., Ogura Y., Konomi H., Sugitani A., Tanaka M. (2003). Appleby Operation for Pancreatic Body-Tail Carcinoma: Report of Three Cases. Surg. Today.

[19] Konishi M., Kinoshita T., Nakagori T., Inoue K., Oda T., Kimata T., Kikuchi H., Ryu M. (2000). Distal pancreatectomy with resection of the celiac axis and reconstruction of the hepatic artery for carcinoma of the body and tail of the pancreas. J. Hepato Biliary Pancreat. Surg..

[20] Mizutani S., Shioya T., Maejima K., Komine O., Yoshino M., Hoshino A., Ogata M., Watanabe M., Yanagimoto K., Shibuya T. (2009). Two successful curative operations using stomach-preserving distal pancreatectomy with celiac axis resection for the treatment of locally advanced pancreatic body cancer. J. Hepato Biliary Pancreat. Surg..

[21] Baumgartner J.M., Krasinskas A., Daouadi M., Zureikat A., Marsh W., Lee K., Bartlett D., Moser A.J., Zeh H.J. (2012). Distal Pancreatectomy with En Bloc Celiac Axis Resection for Locally Advanced Pancreatic Adenocarcinoma Following Neoadjuvant Therapy. J. Gastrointest. Surg..

[22] Wu X., Tao R., Lei R., Han B., Cheng D., Shen B., Peng C. (2010). Distal Pancreatectomy Combined with Celiac Axis Resection in Treatment of Carcinoma of the Body/Tail of the Pancreas: A Single-Center Experience. Ann. Surg. Oncol..

[23] Yamamoto Y., Sakamoto Y., Ban D., Shimada K., Esaki M., Nara S., Kosuge T. (2012). Is celiac axis resection justified for T4 pancreatic body cancer?. Surgery.

[24] Appleby L.H. (1953). The coeliac axis in the expansion of the operation for gastric carcinoma. Cancer.

[25] Okushiba S., Morikawa T., Kondo S., Ambo Y., Tanaka E., Katoh H., Hirano S. (2003). Results of radical distal pancreatectomy with en bloc resection of the celiac artery for locally advanced cancer of the pancreatic body. Langenbeck’s Arch. Surg..

[26] Rebelo A., Büdeyri I., Heckler M., Partsakhashvili J., Ukkat J., Ronellenfitsch U., Michalski C.W., Kleeff J. (2020). Systematic review and meta-analysis of contemporary pancreas surgery with arterial resection. Langenbeck’s Arch. Surg..

[27] Latona J.A., Lamb K.M., Pucci M.J., Maley W.R., Yeo C.J. (2016). Modified Appleby Procedure with Arterial Reconstruction for Locally Advanced Pancreatic Adenocarcinoma: A Literature Review and Report of Three Unusual Cases. J. Gastrointest. Surg..

[28] Hirano S., Kondo S., Hara T., Ambo Y., Tanaka E., Shichinohe T., Suzuki O., Hazama K. (2007). Distal pancreatectomy with en bloc celiac axis resection for locally advanced pancreatic body cancer: Long-term results. Ann. Surg..

[29] Tanaka E., Hirano S., Tsuchikawa T., Kato K., Matsumoto J., Shichinohe T. (2011). Important technical remarks on distal pancreatectomy with en-bloc celiac axis resection for locally advanced pancreatic body cancer (with video). J. Hepato Biliary Pancreat. Sci..

[30] Klompmaker S., Peters N.A., Van Hilst J., Bassi C., Boggi U., Busch O.R., Niesen W., Van Gulik T.M., Javed A.A., Kleeff J. (2019). Outcomes and Risk Score for Distal Pancreatectomy with Celiac Axis Resection (DP-CAR): An International Multicenter Analysis. Ann. Surg. Oncol..

[31] Moher D., Liberati A., Tetzlaff J., Altman D.G. (2009). Preferred reporting items for systematic reviews and meta-analyses: The PRISMA statement. BMJ.

[32] Dindo D., Demartines N., Clavien P.-A. (2004). Classification of Surgical Complications: A new proposal with evaluation in a cohort of 6336 patients and results of a survey. Ann. Surg..

[33] Bassi C., Marchegiani G., Dervenis C., Sarr M., Abu Hilal M., Adham M., Allen P., Andersson R., Asbun H.J., Besselink M.G. (2017). The 2016 update of the International Study Group (ISGPS) definition and grading of postoperative pancreatic fistula: 11 Years After. Surgery.

[34] Panwar R., Pal S. (2017). The International Study Group of Pancreatic Surgery definition of delayed gastric emptying and the effects of various surgical modifications on the occurrence of delayed gastric emptying after pancreatoduodenectomy. Hepatobiliary Pancreat. Dis. Int..

[35] Ottawa Hospital Research Institute. http://www.ohri.ca/programs/clinical_epidemiology/oxford.asp.

[36] Peters N.A., Javed A.A., Cameron J.L., Makary M.A., Hirose K., Pawlik T.M., He J., Wolfgang C.L., Weiss M.J. (2016). Modified Appleby Procedure for Pancreatic Adenocarcinoma: Does Improved Neoadjuvant Therapy Warrant Such an Aggressive Approach?. Ann. Surg. Oncol..

[37] Malinka T., Timmermann L., Klein F., Geisel D., Pratschke J., Bahra M. (2019). Is there a Role for the Appleby Procedure in 2020? Results from a Matched-Pair-Analysis. Anticancer Res..

[38] Storkholm J.H., Burgdorf S.K., Hansen C.P. (2020). Distal pancreas-coeliac axis resection with preoperative selective embolization of the coeliac axis: A single high-volume centre experience. Langenbecks Arch. Surg..

[39] Beane J.D., House M.G., Pitt S.C., Kilbane E.M., Hall B.L., Parmar A.D., Riall T.S., Pitt H.A. (2015). Distal pancreatectomy with celiac axis resection: What are the added risks?. HPB.

[40] Takahashi Y., Kaneoka Y., Maeda A., Isogai M. (2011). Distal Pancreatectomy with Celiac Axis Resection for Carcinoma of the Body and Tail of the Pancreas. World J. Surg..

[41] Okada K.-I., Kawai M., Tani M., Hirono S., Miyazawa M., Shimizu A., Kitahata Y., Yamaue H. (2013). Surgical strategy for patients with pancreatic body/tail carcinoma: Who should undergo distal pancreatectomy with en-bloc celiac axis resection?. Surgery.

[42] Yamamoto T., Satoi S., Kawai M., Motoi F., Sho M., Uemura K.-I., Matsumoto I., Honda G., Okada K.-I., Akahori T. (2018). Is distal pancreatectomy with en-bloc celiac axis resection effective for patients with locally advanced pancreatic ductal adenocarcinoma?—Multicenter surgical group study. Pancreatology.

[43] Hishinuma S., Ogata Y., Tomikawa M., Ozawa I. (2007). Stomach-Preserving Distal Pancreatectomy with Combined Resection of the Celiac Artery: Radical Procedure for Locally Advanced Cancer of the Pancreatic Body. J. Gastrointest. Surg..

[44] Ham H., Kim S.G., Kwon H.J., Ha H., Choi Y.Y. (2015). Distal pancreatectomy with celiac axis resection for pancreatic body and tail cancer invading celiac axis. Ann. Surg. Treat. Res..

[45] Sugiura T., Okamura Y., Ito T., Yamamoto Y., Uesaka K. (2016). Surgical Indications of Distal Pancreatectomy with Celiac Axis Resection for Pancreatic Body/Tail Cancer. World J. Surg..

[46] Ocuin L.M., Miller-Ocuin J.L., Novak S.M., Bartlett D.L., Marsh J.W., Tsung A., Lee K.K., Hogg M.E., Zeh H.J., Zureikat A.H. (2016). Robotic and open distal pancreatectomy with celiac axis resection for locally advanced pancreatic body tumors: A single institutional assessment of perioperative outcomes and survival. HPB.

[47] Sato T., Saiura A., Inoue Y., Takahashi Y., Arita J., Takemura N. (2016). Distal Pancreatectomy with En Bloc Resection of the Celiac Axis with Preservation or Reconstruction of the Left Gastric Artery in Patients with Pancreatic Body Cancer. World J. Surg..

[48] Nakamura T., Hirano S., Noji T., Asano T., Okamura K., Tsuchikawa T., Murakami S., Kurashima Y., Ebihara Y., Nakanishi Y. (2016). Distal Pancreatectomy with en Bloc Celiac Axis Resection (Modified Appleby Procedure) for Locally Advanced Pancreatic Body Cancer: A Single-Center Review of 80 Consecutive Patients. Ann. Surg. Oncol..

[49] Li B., Zhou Y.-M., Zhang X.-F., Li X.-D., Liu X.-B., Wu L.-P. (2014). Distal pancreatectomy with en bloc celiac axis resection for pancreatic body-tail cancer: Is it justified?. Med. Sci. Monit..

[50] Wang C., Wu H., Xiong J., Zhou F., Tao J., Liu T., Zhao G., Gou S. (2008). Pancreaticoduodenectomy with Vascular Resection for Local Advanced Pancreatic Head Cancer: A Single Center Retrospective Study. J. Gastrointest. Surg..

[51] Xue H.-Z., Shen Q., Jiang Q.-F., Tian Y.-W., Yu M., Jia J.-K. (2018). Appleby operation for carcinoma of the body and tail of the pancreas. J. Cancer Res. Ther..

[52] Ueda A., Sakai N., Yoshitomi H., Furukawa K., Takayashiki T., Kuboki S., Takano S., Suzuki D., Kagawa S., Mishima T. (2019). Is hepatic artery coil embolization useful in distal pancreatectomy with en bloc celiac axis resection for locally advanced pancreatic cancer?. World J. Surg. Oncol..

[53] Jing W., Zhu G., Hu X., Jing G., Shao C., Zhou Y., He T., Zhang Y. (2012). Distal Pancreatectomy with En Bloc Celiac Axis Resection for the Treatment of Locally Advanced Pancreatic Body and Tail Cancer. Hepatogastroenterology.

[54] Schmocker R.K., Ms M.J.W., Ding D., Beckman M.J., Javed A.A., Cameron J.L., Lafaro K.J., Burns W.R., Weiss M.J., He J. (2020). An Aggressive Approach to Locally Confined Pancreatic Cancer: Defining Surgical and Oncologic Outcomes Unique to Pancreatectomy with Celiac Axis Resection (DP-CAR). Ann. Surg. Oncol..

[55] Ramia J.M., De Vicente E., Pardo F., Sabater L., Lopez-Ben S., Quijano M.Y., Villegas T., Blanco-Fernandez G., Diez-Valladares L., Lopez-Rojo I. (2020). Preoperative hepatic artery embolization before distal pancreatectomy plus celiac axis resection does not improve surgical results: A Spanish multicentre study. Surgery.

[56] Mittal A., De Reuver P.R., Shanbhag S., Staerkle R.F., Neale M., Thoo C., Hugh T.J., Gill A.J., Samra J.S. (2015). Distal pancreatectomy, splenectomy, and celiac axis resection (DPS-CAR): Common hepatic arterial stump pressure should determine the need for arterial reconstruction. Surgery.

[57] Makary M., Fishman E., Cameron J. (2005). Resection of the Celiac Axis for Invasive Pancreatic Cancer. J. Gastrointest. Surg..

[58] Colombo P.-E., Quenet F., Alric P., Mourregot A., Neron M., Portales F., Rouanet P., Carrier G. (2021). Distal Pancreatectomy with Celiac Axis Resection (Modified Appleby Procedure) and Arterial Reconstruction for Locally Advanced Pancreatic Adenocarcinoma After FOLFIRINOX Chemotherapy and Chemoradiation Therapy. Ann. Surg. Oncol..

[59] Nigri G.R., Rosman A.S., Petrucciani N., Fancellu A., Pisano M., Zorcolo L., Ramacciato G., Melis M. (2010). Metaanalysis of trials comparing minimally invasive and open distal pancreatectomies. Surg. Endosc..

[60] Yu X., Li J., Fu D., Di Y., Yang F., Hao S., Jin C. (2014). Benefit from synchronous portal-superior mesenteric vein resection during pancreaticoduodenectomy for cancer: A meta-analysis. Eur. J. Surg. Oncol..

[61] McMillian N.N. (2020). NCCN Guidelines Panel Disclosures.

[62] Smyth E.C., Verheij M., Allum W., Cunningham D., Cervantes A., Arnold D. (2016). Gastric cancer: ESMO Clinical Practice Guidelines for diagnosis, treatment and follow-up. Ann. Oncol..

[63] Benson A.B., Venook A.P., Al-Hawary M.M., Arain M.A., Chen Y.-J., Ciombor K.K., Cohen S., Cooper H.S., Deming D., Garrido-Laguna I. (2020). NCCN Guidelines Insights: Rectal Cancer, Version 6.2020. J. Natl. Compr. Cancer Netw..

[64] Glynne-Jones R., Wyrwicz L., Tiret E., Brown G., Rödel C., Cervantes A., Arnold D. (2017). Rectal cancer: ESMO Clinical Practice Guidelines for diagnosis, treatment and follow-up. Ann. Oncol..

[65] Ravikumar R., Sabin C., Abu Hilal M., Bramhall S., White S., Wigmore S., Imber C.J., Fusai G. (2014). Portal Vein Resection in Borderline Resectable Pancreatic Cancer: A United Kingdom Multicenter Study. J. Am. Coll. Surg..

[66] Bell R., Ao B.T., Ironside N., Bartlett A., Windsor J.A., Pandanaboyana S. (2017). Meta-analysis and cost effective analysis of portal-superior mesenteric vein resection during pancreatoduodenectomy: Impact on margin status and survival. Surg. Oncol..

[67] Ferrone C.R., Marchegiani G., Hong T.S., Ryan D.P., Deshpande V., McDonnell E.I., Sabbatino F., Santos D.D., Allen J.N., Blaszkowsky L.S. (2015). Radiological and Surgical Implications of Neoadjuvant Treatment with FOLFIRINOX for Locally Advanced and Borderline Resectable Pancreatic Cancer. Ann. Surg..

[68] Yoshiya S., Fukuzawa K., Inokuchi S., Kosai-Fujimoto Y., Sanefuji K., Iwaki K., Motohiro A., Itoh S., Harada N., Ikegami T. (2019). Efficacy of Neoadjuvant Chemotherapy in Distal Pancreatectomy with En Bloc Celiac Axis Resection (DP-CAR) for Locally Advanced Pancreatic Cancer. J. Gastrointest. Surg..

[69] Artinyan A., Anaya D.A., McKenzie S., Ellenhorn J.D.I., Kim J. (2010). Neoadjuvant therapy is associated with improved survival in resectable pancreatic adenocarcinoma. Cancer.

[70] Jang J.-Y., Han Y., Lee H., Kim S.-W., Kwon W., Lee K.-H., Oh D.-Y., Chie E.K., Lee J.M., Heo J.S. (2018). Oncological Benefits of Neoadjuvant Chemoradiation with Gemcitabine Versus Upfront Surgery in Patients with Borderline Resectable Pancreatic Cancer. Ann. Surg..

[71] Pillay B., Wootten A.C., Crowe H., Corcoran N., Tran B., Bowden P., Crowe J., Costello A.J. (2016). The impact of multidisciplinary team meetings on patient assessment, management and outcomes in oncology settings: A systematic review of the literature. Cancer Treat. Rev..

[72] Mercantini P., Lucarini A., Mazzuca F., Osti M.F., Laghi A. (2020). How technology can help in oncologic patient management during COVID-19 outbreak. Eur. J. Surg. Oncol..

