# Supplementary material for: Distal Pancreatectomy with Celiac Axis Resection: Systematic Review and Meta-Analysis
Source: Cancers (Basel). 2021 Apr 19;13(8):1967. doi: 10.3390/cancers13081967 (PMC8073522; doi:10.3390/cancers13081967)
Supplement: Supplementary file 1 [file cancers-13-01967-s001.zip › cancers-1159184-SI.pdf]

**SUPPLEMENTARY MATERIAL**

**Table S1.** Full literature search performed on online databases

| **Keyword used and combined for the search*** | **PubMed**** | **Scopus**** | **WOS**** |
|---|---|---|---|
| DP-CAR | 69 | 76 | 62 |
| Celiac axis resection | 844 | 403 | 342 |
| Distal pancreatectomy | 4109 | 4998 | 3901 |
| Pancreatic Carcinoma | 7347 | 40013 | 29596 |
| Appleby | 2190 | 334 | 317 |
| Arterial resection pancreatic | 2056 | 987 | 706 |

* systematic literature search using the specified databases was performed in December 2020.

**number of results

**Table S2.** Critical appraisal of included studies using Newcastle Ottawa scale. **Stars number**

| **Study, year** | Selection[a] | Comparability[b] | Outcome[c] | Total |
|---|---|---|---|---|
| *Beane, 2015* | 4 | 0 | 1 | 5 |
| *Ham, 2015* | 4 | 0 | 2 | 6 |
| *Hishinuma, 2007* | 4 | 0 | 1 | 5 |
| *Malinka, 2020* | 4 | 0 | 2 | 6 |
| *Okada, 2012* | 4 | 0 | 2 | 6 |
| *Peters, 2016* | 4 | 1 | 1 | 6 |
| *Storkholm, 2020* | 4 | 0 | 2 | 6 |
| *Sugiura, 2017* | 4 | 0 | 2 | 6 |
| *Takahashi, 2011* | 4 | 0 | 1 | 5 |
| *Wu, 2010* | 4 | 0 | 2 | 6 |
| *Yamamoto, 2017* | 4 | 1 | 2 | 7 |

[a] Maximum 4 stars; [b] Maximum 2 stars; [c] Maximum 3 stars.
